# Coping with Uncertainty in Everyday Situations (CUES©) to address intolerance of uncertainty in autistic children: study protocol for an intervention feasibility trial

**DOI:** 10.1186/s13063-019-3479-0

**Published:** 2019-06-27

**Authors:** Jacqui Rodgers, Jane Goodwin, Jeremy R. Parr, Victoria Grahame, Catharine Wright, John Padget, Deborah Garland, Malcolm Osborne, Marie Labus, Ashleigh Kernohan, Mark Freeston

**Affiliations:** 1 Institute of Neuroscience, Sir James Spence Institute, Newcastle University, Royal Victoria Infirmary, Queen Victoria Road, Newcastle, NE1 4LP UK; 2 Complex Neurodevelopmental Disorders Service, Northumberland Tyne and Wear NHS Foundation Trust, Walkergate Park Hospital, Walkergate Park, NE6 4QD UK; 3 Child and Adolescent Mental Health Service, Northumbria Healthcare NHS Foundation Trust, Albion Rd Resource Centre, North Shields, NE29 0HG UK; 4 Sir James Spence Institute, Newcastle University, Royal Victoria Infirmary, Queen Victoria Road, Newcastle, NE1 4LP UK; 5 South Tyneside’s Kids And Young Adults Klub - Special needs support group (KAYAKS), South Shields, NE33 4UG UK; 6 Research and Enterprise Services, Faculty of Medical Sciences, Newcastle University, The Medical School, Framlington Place, Newcastle upon Tyne, NE2 4HH UK; 7 Institute of Health & Society Newcastle University Baddiley-Clark Building, Richardson Road, Newcastle upon Tyne, NE2 4AX UK; 8 School of Psychology, Newcastle University, 4th Floor Ridley Building, Newcastle upon Tyne, NE1 7RU UK

**Keywords:** Anxiety, Intolerance of uncertainty, Parent group, Intervention study, Autism

## Abstract

**Background:**

Anxiety is a common diagnosis in children with autism spectrum disorder (ASD). One key mechanism underlying anxiety is intolerance of uncertainty, which is a tendency to react negatively on an emotional, cognitive, and behavioural level to uncertain situations and events. We developed the first intervention programme specifically targeting intolerance of uncertainty in children with ASD: Coping with Uncertainty in Everyday Situations (CUES). CUES is a parent group intervention providing parents of children with ASD with strategies to increase tolerance to uncertainty for their children in everyday situations. The principal aims of the current study are: 1) evaluate the acceptability and feasibility of delivering CUES to parents who have a child with ASD and anxiety; and 2) inform the design of a fully powered trial.

**Method:**

This is a feasibility and acceptability single-blind pilot randomised controlled trial comparing CUES (intervention) to a brief psychoeducation, emotional literacy, and relaxation programme (enhanced services as usual). Participants will be assessed at baseline and followed-up immediately post-treatment, and at 12 and 26 weeks post-treatment. Parents who have a child with ASD and anxiety (aged 6–16 years) will be invited to take part in the study and written parental informed consent and child assent will be obtained. Participants will be recruited from the National Health Service mental health teams in the UK. Sixty participants will be randomised to either intervention or enhanced services as usual in a 1:1 ratio.

**Discussion:**

The present study will provide evidence on the acceptability of the CUES intervention to parents and children, and the feasibility of recruitment and delivery to inform the design and sample size for a full-scale randomised controlled trial. Qualitative data will be obtained to understand how feasible CUES is for families, and the experiences of participants regarding their experiences of the intervention.

**Trial registration:**

ISRCTN, ISRCTN10139240. Registered on 14 May 2018.

**Electronic supplementary material:**

The online version of this article (10.1186/s13063-019-3479-0) contains supplementary material, which is available to authorized users.

## Background

Mental health, and specifically anxiety, is amongst the top five research priorities for future autism spectrum disorder (ASD) research [1]. Approximately 50% of children with ASD experience high anxiety, congruent with an anxiety disorder [2, 3], which significantly impacts on everyday life for them and their families. Anxiety is a major risk factor for mental health difficulties in adulthood, including suicidal thoughts and behaviours [4]. When present, anxiety in individuals with ASD is often complex, encompassing features of a range of concurrent anxiety disorders, as well as atypical presentations [5, 6]. Therefore, treatment targeting the underlying mechanisms rather than specific symptoms is likely to be necessary and may be more effective. One key mechanism is intolerance of uncertainty (IU). IU is a ‘broad dispositional risk factor for the development and maintenance of clinically significant anxiety’ [7]. It involves the ‘tendency to react negatively on an emotional, cognitive, and behavioural level to uncertain situations and events’ [8].

Studies of people with ASD suggest that IU is a transdiagnostic construct associated with a range of anxiety disorders [9–13]. Importantly, intervention studies with neurotypical individuals suggest that a reduction in IU is associated with a reduction in anxiety and improvements in everyday functioning (see, for example, [14]). The role of IU in anxiety in typically developing adolescents [15–17] and children [18, 19] has been increasing, and it is argued that cognitive behavioural treatments, which emphasise treating the cognitive process rather than the cognitive content of anxiety, specifically by aiming to increase the tolerance of patients for uncertainty, achieve more sustainable change [20, 21]. Research has confirmed the utility of such protocols in reducing anxiety in adults and children without ASD [22–25].

Recently, IU has been associated with some of the core characteristics of ASD [26–28]. Restricted and repetitive behaviours, such as insistence on sameness, inflexible adherence to routines, and difficulty tolerating change, have been linked with anxiety in ASD since the earliest descriptions of the disorder [29] and bear a conceptual resemblance to IU; that is, avoidance of unexpected events and desire to make life as predictable as possible [30]. There is also evidence that IU has a central role in the relationship between ASD and anxiety. Boulter et al. [11] reported significant relationships between IU and anxiety in children with ASD, and their results suggested that, compared with matched neurotypical controls, IU levels account for the increased anxiety observed in children with ASD. Wigham et al. [10] examined the role that IU has in pathways between sensory processing difficulties, anxiety, and restricted and repetitive behaviours in ASD. These relationships were mediated by IU, indicating the important role IU may have in the interaction between anxiety and ASD traits. Chamberlain et al. [12] reported associations between shared neurobehavioural mechanisms in ASD and anxiety, indicating specific avenues for intervention targeting IU. Rodgers et al. [31] developed and validated a child self-report and parent-report measure of anxiety for children with ASD (the ASC-ASD). Using factor analytic techniques, Rodgers identified four reliable anxiety subscales, including an uncertainty scale. Hodgson et al. [32] undertook focus groups with parents of children with ASD exploring the concept of IU. Parents differentiated IU from dislike of change and from fear, discussed examples of IU and its impact on their children, and suggested that IU is a recognisable and important construct associated with anxiety distinguishable from but related to features of ASD. Although many of the studies come from the Newcastle group, in an independent multisite study Keefer et al. [9] demonstrated that high levels of pretreatment IU significantly predicted poorer treatment response in a group of children with ASD receiving treatment for anxiety. Neil et al. [33] further replicated that IU is a relevant construct related to sensory sensitivities and anxiety in children with ASD. Finally, Kerns et al. [34] in a discussion of the differential diagnosis of anxiety disorders in ASD report that fears associated with uncertainty may be an important mechanism in the development and maintenance of anxiety in ASD. In conclusion, this evidence indicates that IU appears to be an important mechanism in the development and maintenance of anxiety for children with ASD and an appropriate target for intervention.

Therefore, we developed the first intervention programme specifically targeting IU in children with ASD: Coping with Uncertainty in Everyday Situations (CUES) [5]. CUES is an 8-week parent group intervention that provides parents of children with ASD with strategies to increase tolerance of uncertainty for their children in everyday situations. Our CUES development project [5] provided preliminary evidence that CUES is feasible and acceptable to families, that parents found it helpful to work with clinicians to develop a range of strategies to tackle uncertainty in everyday contexts that they can support their child to utilise, and parents and children reported beneficial effects on everyday functioning. We now plan to conduct a feasibility and acceptability randomised pilot trial of CUES. The SPIRIT checklist is included as Additional file 1.

## Methods/design

### Trial design

This is a feasibility and acceptability pilot randomised controlled trial of a parent group-based intervention aimed at increasing tolerance to uncertainty in children with ASD: Coping with Uncertainty in Everyday Situations (CUES) [5].

### Aims

We aim to: 1) ascertain whether CUES is feasible to deliver through UK National Health Service (NHS) services by trained therapists, and is acceptable and well received by families; 2) establish the rate of referrals from child mental health teams in two NHS Trusts; 3) record the proportion of families who agree to participate and the proportion who complete all sessions, and to explore suitable methods of data collection and outcomes in relation to health services costs and costs to families of children with IU; 4) investigate whether treatment fidelity can be maintained across therapists; 5) record response rates for completion of outcome measures; 6) obtain participant feedback on the programme and valued outcome measures; 7) determine the acceptability/credibility of our active control group procedures; 8) investigate whether the families who participate in CUES have greater reduction in real-life IU and anxiety than children receiving enhanced services as usual; 9) describe variability in responses to CUES in children referred from NHS services; and 10) monitor whether any treatment effects persist 6 months post-treatment (primary endpoint).

### Setting and participants

Sixty parent participants will be recruited via NHS services (including diagnostic clinics and Child and Adolescent Mental Health Services). This number is based on recommendations for good practice in feasibility studies [35]. Parents are eligible for study entry if their child meets the following criteria: 1) a diagnosis of ASD; 2) aged 6–16 years; 3) experiences some anxiety; 4) no intellectual disability or mild-to-moderate intellectual disability; and 5) have sufficient language ability to complete appropriate outcome measures (with assistance if required).

Parents themselves will have sufficient spoken and written English to provide written informed consent, complete assessments, and participate in the intervention. Parents are not eligible for study entry if their child meets the following criteria: 1) no diagnosis of ASD diagnosis; 2) experiences severe and complex anxiety disorder (based on the clinical judgement of clinicians); 3) has severe intellectual disability; or 4) has complex health conditions.

Furthermore, children of parents who have significant mental health difficulties will not be eligible to participate.

### Participant identification and recruitment process

**Fig. 1:**
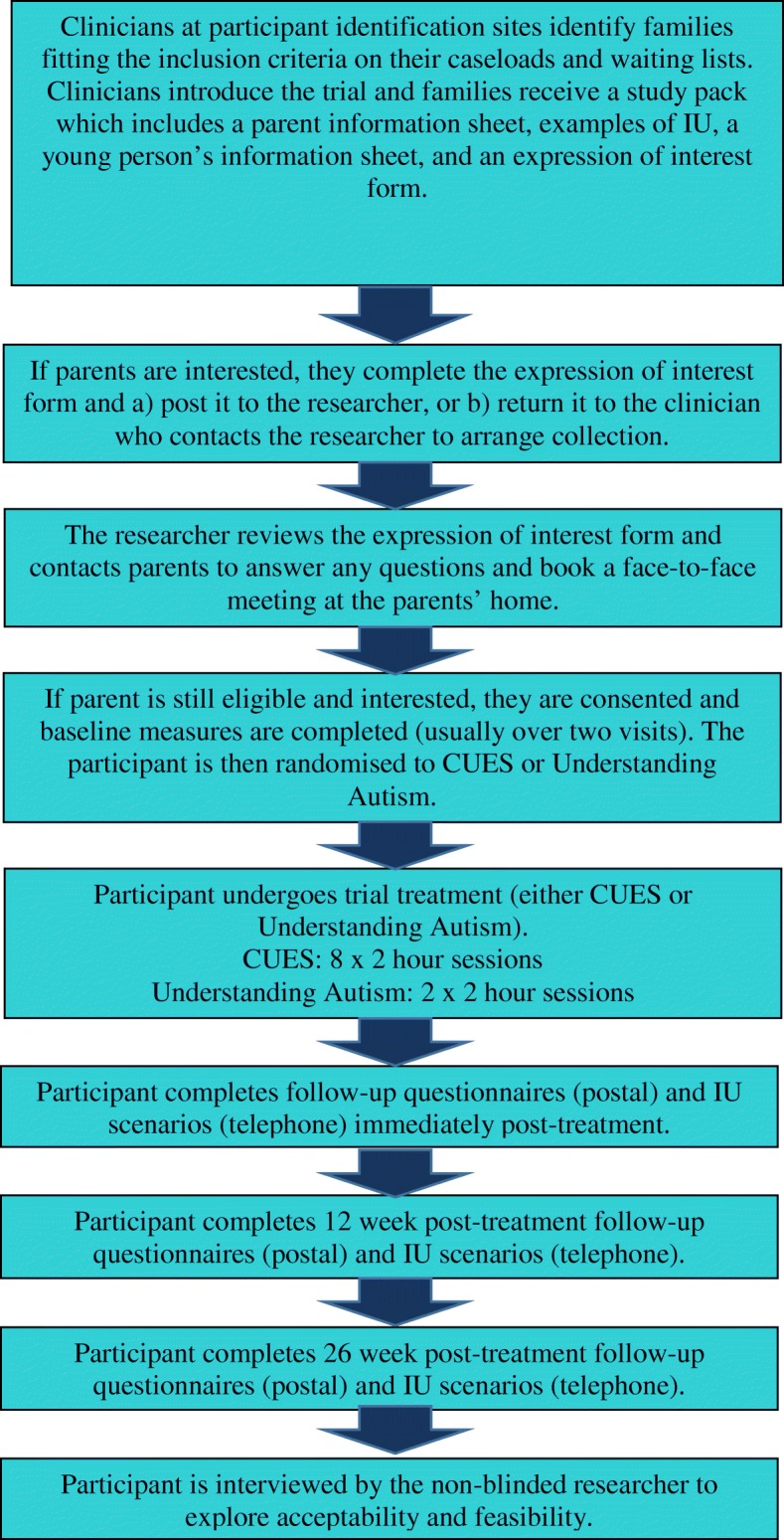
Participant identification, recruitment, and follow-up procedure. CUES Coping with Uncertainty in Everyday Situations, IU intolerance of uncertainty

Families will be identified via NHS services. At NHS services, clinicians at the participating identification centres will be provided with information about IU to ensure familiarity with the construct to enable them to introduce the study to families. Clinicians will identify children fitting the inclusion criteria, discuss the study with them, and if interested give them study packs (Fig. 1). Interested parents will complete an expression of interest form that will be sent to the research team. The research team will then contact parents to arrange a face-to-face meeting to discuss the study, what participation involves, answer any questions they may have, and arrange written informed consent. Baseline data will be collected at this point, prior to randomisation.

Information provided to participants makes it clear that they do not have to participate in the trial and their decision will not affect their treatment. The participant can withdraw from the study at any time, which will not affect any further NHS treatment or services.

### Treatment

#### Intervention arm: CUES

CUES is an 8-week manualised programme [5] that provides parents of children with ASD the strategies to increase tolerance to uncertainty in everyday situations in their children. It was devised in partnership with parents. The key goal of the programme is to increase the child’s tolerance to uncertainty, reduce negative beliefs about uncertainty, and develop a more flexible approach to uncertainty. A parent-mediated intervention is appropriate because it provides parents with strategies that they can utilise with their child across a range of everyday contexts. It also supports generalisation of these strategies outside of the clinic setting, as well as countering well-meaning, understandable but ultimately unhelpful/counter-productive strategies used by parents to try to build certainty around the child, when certainty is not realistically possible. Thus, working with parents increases the translational potential of the intervention.

Parents will attend the programme, with two therapists with expertise in ASD facilitating each group. Each group will include approximately ten parents and sessions will take place weekly for 8 weeks. Each session will be 2 h in duration. ‘At home’ activities will be set each week for parents and children to complete between sessions. The programme begins with a focus on the development of understanding of uncertainty in everyday life, the nature and impact of IU, and promotes the use of strategies to flexibly manage IU across a range of settings. The intervention helps parents to recognise IU and identify potential developmental and environmental factors that may trigger IU for their child. It further teaches parents to plan and use appropriate strategies aimed at increasing their child’s tolerance of uncertainty. Each parent will be provided with weekly materials and individual support to identify strategies to address a chosen target IU situation. This target situation is the focus for parents to practise the new strategies with their child, thus ensuring that strategies are individually tailored for each child and are developmentally appropriate. The group situation provides extensive opportunities for mutual learning and support. The CUES intervention has incorporated and synthesised components of existing good practice in relation to anxiety treatment for children with ASD. It provides the opportunity for parents to develop an understanding of IU and its impact, to try out various strategies, and provides opportunities for discussion, mutual support and sharing of ideas, and experiences and strategies, importantly building parents’ knowledge and confidence to support their child at home to develop a more flexible approach to uncertainty.

Therapists delivering CUES are trained in the skills needed to deliver the programme through two training sessions (5 h in total). To ensure the intervention is being delivered as intended, with high fidelity to the manual, the principal investigator (JR) and the clinical lead (VG) will undertake supervision and monitoring of the quality of parent group sessions by video, providing timely feedback to group leaders. Weekly supervision of the therapists will be conducted by JR and VG. Treatment fidelity will be maintained and investigated from multiple standpoints, including supervisor feedback, and formally by observer ratings of recordings of the group sessions.

#### Enhanced services as usual arm: Understanding Autism

Understanding Autism will comprise two parent group sessions. Each session will be 2 h in duration. Parent group-based interventions provide parents with the opportunity not only to develop knowledge and skills in relation to the treatment target (in this case IU), but also provide opportunities for discussion, mutual support, and sharing of ideas, a process which parents tell us they value. Given the feasibility and acceptability aims of the current study, we were concerned that offering families in the control arm no opportunity to meet together as a group might significantly bias favourable responses in relation to acceptability towards the CUES intervention arm. In addition, treatment as usual is highly variable and thus the introduction of a two-session programme for the enhanced services as usual arm enables us to provide parents with some common content, and credibility/expectancy will be rated for both arms, whereas services as usual cannot be rated; this creates a greater level of equipoise than treatment as usual. There is currently no equivalent eight-session parent support group available to parents of children with ASD within this age range, and parents in the control arm receive a two-session Understanding Autism programme. This enables parents randomised to the control arm to have experience of a parent group setting. Both arms will continue to receive treatment as usual, which will be recorded on the demographics form.

The group will focus on psychoeducation, social communication, repetitive behaviours, and making and keeping friends. There are opportunities for parents to engage in group discussion regarding their experiences, such as the ASD diagnostic process.

Group leaders for Understanding Autism have extensive knowledge on ASD and have a track record of involvement in research and community outreach. The materials were developed in collaboration with the research team. They will receive weekly supervision with the principal investigator. Further, to ensure Understanding Autism is being delivered as intended (without reference to anxiety or IU), the principal investigator (JR) will monitor the quality of parent group sessions by video, providing timely feedback to group leaders. Fidelity will be formally assessed by an independent rater utilising a fidelity checklist and recordings of the group sessions. The control group will not have access to the intervention after the trial.

### Strategies to maintain adherence

A number of strategies will be utilised to improve adherence in terms of attendance at the intervention sessions and completion of the follow-up assessments. These includes written information about the venues for the sessions, in the case of absence from a session the materials for the session are posted to the participant to be received during the week of absence and a telephone call arranged between one of the therapists and the parents to ensure they have received the materials, determine if they have any questions, and encourage attendance at the next session. Parents are sent reminder emails, followed up with a telephone call if follow-up questionnaires have not been received. All parents receive regular newsletters about the study, along with seasonal greetings cards to encourage continued participation.

### Harm

Based on the development study for CUES [5] adverse events or unintended effects are not anticipated. However, if a clear safeguarding concern arises this will trigger an immediate response. The staff delivering the programmes are employees of the sponsoring Trust and will follow Trust protocols. In addition, therapists will receive weekly supervision from the principal investigator, on the same day as the intervention session takes place, and reporting of any unintended effects or adverse events is included as a standard item on the supervisory agenda. If any such events are identified, they will be discussed with the clinical lead which will result in action appropriate to the situation following the Standard Operating Procedures of the sponsor (Northumberland Tyne and Wear NHS Foundation Trust (NTW)), which are compliant with Good Clinical Practice (GCP) and Health Research Authority (HRA) guidelines for safety reporting. All such events will be recorded in the site file.

### Confidentiality and access to data

All the data collected as part of the study will be kept strictly confidential and in accordance with the General Data Protection Regulation (GDPR). Participants are provided with detailed information relating to confidentiality in the participant Information Sheet. Northumberland Tyne and Wear NHS Foundation Trust (NTW) is the sponsor and will act as the data controller. The study is being led and managed by Newcastle University, who are acting as a data processor. NTW and Newcastle University will keep identifiable information about participants until 3 years after the youngest child in the study reaches 18 years old. The local study team at NTW and Newcastle University will use participants’ names and contact details to contact them about the research study, and make sure that relevant information about the study is recorded for their child’s care, and to oversee the quality of the study. Individuals from NTW, Newcastle University, and regulatory organisations may look at the research records to check the accuracy of the research study. The only people who will have access to information that identifies participants will be people who need to contact them about the study or audit the data collection process. All information will be stored in a secure and locked office, and on a password-protected database. Any information which leaves the hospital or university sites will have names and addresses removed (anonymised) and a unique code will be used. Personal data (name, address, email, telephone number) will be kept after the end of the study so that we are able to contact participants about the findings of the study.

### Randomisation and blinding

Participants are randomised on a 1:1 basis to receive either CUES or Understanding Autism, without stratification. Randomisation occurs online through Sealed Envelope (https://www.sealedenvelope.com/). Participants will be aware of group status due to the nature of the intervention, but the primary outcome assessor will be blind to group allocation although, despite instruction not to do so, participants could unwittingly break blindness if they reveal details about the intervention at post-treatment assessment.

### Procedure

The researcher will meet with the participant at home to explain the study, answer questions, obtain informed consent (and assent from the child), and undertake the baseline characterisation and target uncertain situation interviews. This may be split into two visits depending on logistics (e.g., parents’ availability) and the length of time the interviews take, as this will vary between participants. Prior to the first visit, the assessments in questionnaire format are posted to the parent to complete. These are collected once the participants have been consented at the home visit(s).

### Primary and secondary outcomes

We are aiming to determine whether CUES is feasible to deliver through NHS services by trained therapists, and if it is acceptable and well received by families. Also, we will investigate whether successfully targeting IU will result in an increased tolerance to uncertainty and a downstream reduction in a range of anxiety disorder symptoms, alongside an increase in children’s social participation as evidenced by the respective outcome measures. In addition, we will explore whether participation in CUES will have a beneficial effect on parental wellbeing.

#### Baseline characterisation

- The characteristics and functional abilities of the child with ASD will be measured by the Social Communication Questionnaire (SCQ; [36]) (parent reporting on child). This is a 40-item questionnaire which is used internationally and has high sensitivity and specificity for an ASD diagnosis [36]. We will also use the Vineland Adaptive Behaviour Scales III (VABS III; [37]) (parent reporting on child). The number of items depends on the child’s developmental level. It has demonstrated excellent internal consistency (α = 0.90 to 0.98) and content, construct, and concurrent validity [37].
- The anxiety characteristics of the child with ASD will be measured with the Anxiety Disorders Interview Schedule — Autism Spectrum Addendum (ADIS-ASA; [38]) (parent reporting on child). The number of items depends on the child’s symptoms of anxiety. It is a reliable measurement of comorbidity (intraclass correlation (ICC) = 0.85–0.98; κ =0.67–0.91) as well as ambiguous anxiety-like symptoms (ICC = 0.87–95, κ = 0.77–0.90) in children with ASD. Convergent and discriminant validity were supported for the traditional anxiety symptoms on the ADIS/ASA, whereas convergent and discriminant validity were partially supported for the ambiguous anxiety-like symptoms. We will all interview the parent to obtain information about the child’s current treatment and other health service usage; concurrent life events that may influence mood; perceived impact of anxiety on the child’s education and participation in everyday events; and impact of the child’s anxiety on other members of the family (parent reporting on child).

#### Feasibility and acceptability outcomes

- The outcomes are acceptability of all aspects of the trial (including outcome measures, acceptability of intervention materials and methods, use of strategies outside of sessions, and any perceived benefits after treatment) and feasibility (including experience of recruitment, randomisation, and visits to the home). These will be obtained via parent interview (parent self-report and parent reporting on child) and the Credibility and Expectancy Questionnaire [39] (parent self-report). The CUES group completes a 10-item questionnaire and the Understanding Autism group completes a 6-item questionnaire. This has demonstrated high internal consistency within each factor (α = 0.86) and good test–retest reliability. The expectancy factor predicts outcome on some measures, whereas the credibility factor is unrelated to outcome [39]. We will also conduct interviews with therapists.

#### Main research outcome

- The main research outcome is to target uncertain situations that cause the child to experience anxiety due to IU (parent reporting on child) using a target behaviour rating. Parents will be asked to identify two target real-life situations that might cause their child significant IU (method validated in our pilot work [5]), one situation that their child would like to do but currently cannot do or not do consistently (e.g. playing with friends in the neighbourhood), and one situation that is appropriate and necessary for them to do due to normal developmentally appropriate educational, social participation, or inclusion outcomes (e.g. attending swimming lessons). Through a semistructured interview, parents will be asked what about the situation is uncertain, how often it occurs, the child’s reaction (symptoms and intensity), whether the child worries about the situation in advance, and how it interferes with daily functions and activities for the child and the family. This can include adaptations to family life, the emotional impact, and the impact on relationships (child, siblings, peers, school, family, how they feel about themselves, and development of autonomy). The interview schedule may be an iterative process to ensure the situation involves IU and is appropriate for CUES in that it is current and presents an ongoing rather than a ‘one-off’ situation.
- This assessment protocol is based on that used by The Research Unit on Paediatric Psychopharmacology and Psychosocial Interventions [40] and has been used in previous anxiety and other treatment trials (e.g. [41, 42]). The IU target situations will be rated on a nine-point scale of improvement/deterioration independently by a panel of experienced clinicians. The top 3 points will define ‘a responder’. Arnold et al. [40] report ICC of 0.895 for a panel of five experts.

#### Secondary research outcomes

- IU will be measured with the Intolerance of Uncertainty Scale (IUS-P and IUS-C; [43]) (parent reporting on child and (where possible) child self-report). Both are 12-item questionnaires. Both child (C) and parent (P) measures show acceptable to excellent internal consistency in ASD (α = 0.78 and 0.90, respectively) and typically developing groups (α = 0.76 and 0.91, respectively). We will also use the Intolerance of Uncertainty Scale (IUS-12; [7]) (parent self-report). This is a 12-item questionnaire, which has excellent internal consistency in a clinical sample (α = 0.91) and community sample (α = 0.92). The average inter-item correlation was 0.48 [7].
- Anxiety will be measured by three items. The Screen for Child Anxiety Related Disorders (SCARED; [44]) (parent reporting on child) is a 41-item questionnaire which has demonstrated good internal consistency (α = 0.74–0.93), test-retest reliability (ICC 0.70–0.90), and discriminative validity, both between anxiety and other disorders and within anxiety disorders [44]. The Anxiety Scale for Children — ASD – Parent and Child versions (ASC-ASD; [31]) (parent reporting on child and (where possible) child self-report) both (parent and child versions) have 24 items, and have demonstrated good to excellent internal consistency (α = 0.85–0.91), validity with measures (including sensory processing hypersensitivity, repetitive behaviours, depression, and anxiety), and 1 month test–retest reliability (*r* = 0.84, ICC = 0.84 for parent; *r* = 0.82, ICC = 0.82 for child) [31]. The Depression, Anxiety and Stress Scale (DASS; [45]) (parent self-report) is a 42-item questionnaire. This has acceptable reliability (α = 0.84–0.91), and convergent and discriminant validity [45].
- Participation and enjoyment will be assessed with the Children’s Assessment of Participation and Enjoyment (CAPE; [46]) (child self-report). This is a 55-item questionnaire, which has demonstrated good internal consistency (α = 0.30–0.62, which is expected due to the environmental, family, and child factors that affect participation), test–retest reliability (ICC = 0.64–0.86), and sufficient content and construct validity [46].
- Self-efficacy will be assessed with the Parent self-efficacy [47] (parent self-report). This is a 15-item questionnaire that ask parents to rate their confidence and self-efficacy in relation to behaviours targeted in clinical studies (in this case anxiety and IU-related behaviours). It is widely used for parent-mediated interventions and there are reliability and validity data available.
- Reactions to uncertainty and confidence will be measured using a bespoke questionnaire administered to parents each week of the CUES programme (parent reporting on self and child).
- Resource use will be assessed with a bespoke questionnaire given to parents measuring health resource use associated with IU in their child.
- Time and travel costs will be assessed with a bespoke questionnaire administered to parents measuring the travel costs and travel time associated with managing their child’s IU.

### Data collection and data management

**Fig. 2:**
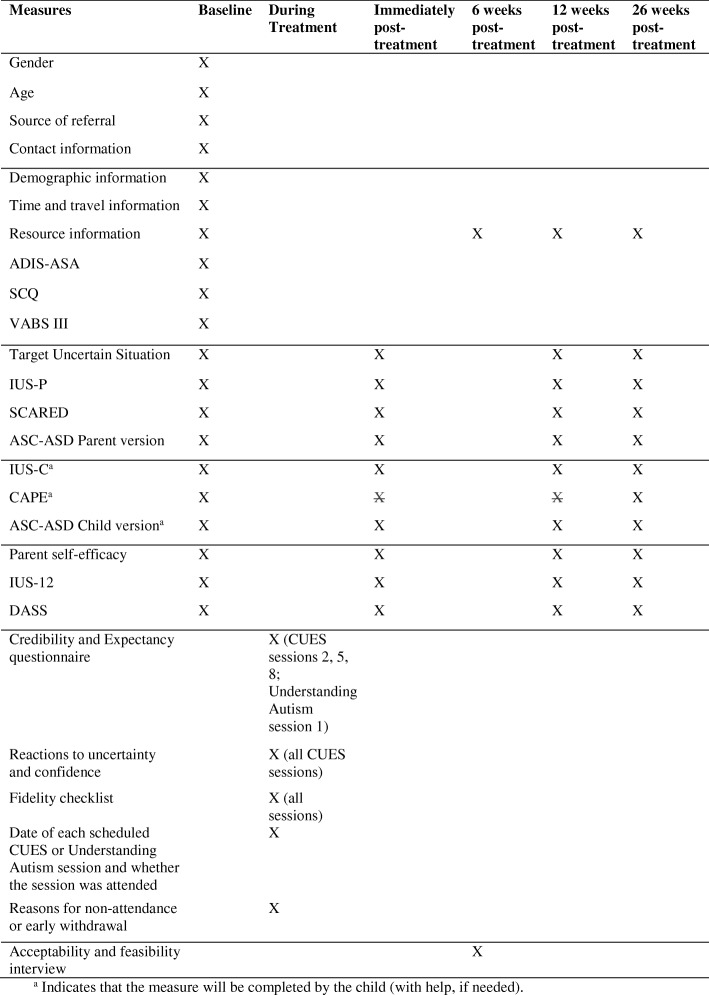
Time points at which measures and data are collected. ADIS-ASA Anxiety Disorders Interview Schedule — Autism Spectrum Addendum, ASC-ASD Anxiety Scale for Children — Autism Spectrum Disorder, CAPE Children’s Assessment of Participation and Enjoyment, CUES Coping with Uncertainty in Everyday Situations, DASS Depression, Anxiety and Stress Scale, IUS-C Intolerance of Uncertainty Scale Child, IUS-P Intolerance of Uncertainty Scale Parent, SCARED Screen for Child Anxiety Related Disorders, SCQ Social Communication Questionnaire, VABS III Vineland Adaptive Behaviour Scales III

Figure 2 details the information collected at different time points. To reduce burden for the family, follow-up assessments using questionnaire measures will be completed by post and the IU scenarios outcome measure will be rated by parents during follow-up telephone semistructured interviews with the blinded researcher. Northumberland Tyne and Wear NHS Foundation Trust may audit the data collected. Data collected on paper assessment tools will be entered onto a secure statistical analysis software system. A unique trial number will be used to identify participants on all paper data collection forms throughout the duration of the trial. No participant-identifiable data will leave the study site. The quality and retention of study data will be the responsibility of the principal investigator. All study data will be retained in accordance with the latest Directive on Good Clinical Practice (2005/28/EC) and local policy. Staff involved in the conduct of the trial, including the principal investigator, Trial Management Group and therapist staff involved in screening and intervention, will have access to the site files. Clinical information shall not be released without the written permission of the participant, except as necessary for monitoring and auditing by the Sponsor, its designee, regulatory authorities, or the REC. The principal investigator and staff involved in this trial may not disclose or use for any purpose, other than performance of the trial, any data, record, or other unpublished, confidential information disclosed to those individuals for the purpose of the trial.

### Planned analyses

Sydes and colleagues [48], who reviewed requirements for data monitoring committees (DMCs), concluded that DMCs are recommended when trials have any of the following features: trials on high-profile topics that are a focus of community concern, that will be used to seek regulatory approval or that are likely to profoundly affect clinical practice; trials with serious safety concerns, unknown risks or that are implemented in vulnerable populations; and trials where independent monitoring is needed because of double-blind treatment assignment or long-term follow-up or because the sponsoring company does not have standard operating procedures. DMCs may not be needed: 1) for trials that are of short duration (where it may not be feasible to convene a DMC in a timely fashion to review the data); 2) for trials with known risks that are minimal; 3) for trials in which the objective is to demonstrate principles (such as in early-phase clinical trials); or 4) for trials on behavioural or administrative issues. In the current case, given the acceptability and feasibility aims where we are constantly monitoring events that could relate to acceptability and feasibility such as nonattendance (for any reason) and, if not informed of a reason, this is followed-up, coupled with the short duration, minimal risks, and the parent-delivery of treatment, it was determined that a DMC was not required. In this case, data management will be discussed at Trial Steering Committee meetings.

As this is a feasibility study, analyses will be mainly descriptive, and no interim analysis will be undertaken. Formal power calculations are not appropriate as the study is not designed to test for a difference between treatments. Feasibility will be explored by examining: 1) the number of families who consented, their attendance, and complete data at 6 months; and 2) that qualitative data support the acceptability of CUES by the majority of parents and clinicians.

We will also undertake some preliminary analysis of treatment effects. As a randomised controlled trial, primary analysis will be based on the intention-to-treat principle with analysis based on groups allocated at randomisation and all randomised families included. The extent of missing data will be assessed. Rates will be calculated as defined and reported with 95% confidence intervals. At baseline and by group, the distribution of all variables will be examined and summarised by measures of central location and spread and reported with 95% confidence intervals. Baseline categorical variables will be tabulated and percentages reported with 95% confidence intervals. The difference in mean change for all outcome measures will be examined between the groups from baseline to each of the two time points with accompanying 95% confidence intervals. Evidence of clustering and/or contamination will be explored. Such results will be interpreted cautiously because of the size of the study and the possible imbalance in prerandomisation baseline covariates. The relationship between baseline covariates and outcome measures will be examined graphically and quantified appropriately depending on their distribution.

While no formal economic evaluation will be undertaken at this stage, questionnaires have been designed in conjunction with patient representatives which will measure resource use associated with managing IU. The data gathered from these questionnaires will provide information about healthcare resource use and personal costs to families which will aid the design of a potential future economic evaluation associated with a full trial. Numbers of families who express interest, continue to treatment, and who complete treatment will be recorded. When families do not participate/complete treatment, the reasons for this will be investigated, if appropriate and where possible, through brief telephone interviews or visits to families. The study team and the health economist will advise on the content of the interview schedule.

When appropriate, interviews will be recorded, transcribed, double-coded, and analysed using thematic analysis [49].

### Dissemination plan

Dissemination of the findings will be undertaken in a number of ways. Co-applicants representing the autism community (DG and MO) will review the findings alongside the Patient and Public Involvement group and other members of the research team and assist with “translating” the main findings and implications for dissemination; a film will be made about the findings and posted on research group websites with links distributed during dissemination events. A series of reports will be written in an accessible and inclusive manner for a lay audience, including reports and newsletters for autistic people and their families, professionals and commissioners, and other agencies. Some versions will be prepared using ‘easyread’ to maximise accessibility and will be available in both written and video format. A feedback event will be hosted to present findings to families. Findings will be presented at academic meetings and written up for open-access peer-reviewed journals. UK multidisciplinary team clinicians will be informed through presentations at relevant professional meetings and conferences. Articles will be written for the professional organisations’ newsletters.

## Discussion

Anxiety affects approximately 50% of children with ASD. It significantly impacts on the everyday life of families and is a major risk factor for mental health difficulties, including suicide, in adulthood. There is evidence to suggest that IU plays a central role in the relationship between ASD and anxiety (e.g. [11]). CUES aims to target IU by providing parents with strategies to manage children’s IU and improve their everyday functioning. Therefore, CUES has the potential to improve psychological outcomes for children with ASD and their families because it provides parents with strategies that they can utilise with their child across a range of everyday contexts.

The present study will provide data on the acceptability of the CUES intervention as well as feasibility trial data to inform the design and sample size for a full-scale trial. In addition, qualitative data will be obtained to understand how feasible CUES may be for families based on participants’ experiences of the intervention.

## Trial status

The first participant was consented to the CUES trial on 19 June 2018. Recruitment is planned to continue until February 2019.

## Additional file

Additional file 1: SPIRIT 2013 checklist: recommended items to address in a clinical trial protocol and related documents. (DOC 120 kb)

## Data Availability

The datasets generated and/or analysed during the current study are available from the corresponding author on reasonable request.

## Abbreviations

ASD: Autism spectrum disorder
CUES: Coping with Uncertainty in Everyday Situations
DMC: Data monitoring committee
IU: Intolerance of uncertainty
NHS: National Health Service
